# Cross-kingdom host shifts of phytomyxid parasites

**DOI:** 10.1186/1471-2148-14-33

**Published:** 2014-02-23

**Authors:** Sigrid Neuhauser, Martin Kirchmair, Simon Bulman, David Bass

**Affiliations:** 1 Institute of Microbiology, Leopold-Franzens University Innsbruck, Technikerstraße 25, 6020 Innsbruck, Austria; 2 Department of Life Sciences, Natural History Museum London, Cromwell Road, London SW7 5BD, UK; 3 The New Zealand Institute for Plant & Food Research Limited, Private Bag 4704, Christchurch 8140, New Zealand

**Keywords:** Plasmodiophorid, Plant pathogen, Evolution, Taxonomy, Host range, *Plasmodiophora*, *Polymyxa*, *Woronina*, *Sorosphaerula*, *Spongospora*

## Abstract

**Background:**

Phytomyxids (plasmodiophorids and phagomyxids) are cosmopolitan, obligate biotrophic protist parasites of plants, diatoms, oomycetes and brown algae. Plasmodiophorids are best known as pathogens or vectors for viruses of arable crops (e.g. clubroot in brassicas, powdery potato scab, and rhizomania in sugar beet). Some phytomyxid parasites are of considerable economic and ecologic importance globally, and their hosts include important species in marine and terrestrial environments. However most phytomyxid diversity remains uncharacterised and knowledge of their relationships with host taxa is very fragmentary.

**Results:**

Our molecular and morphological analyses of phytomyxid isolates–including for the first time oomycete and sea-grass parasites–demonstrate two cross-kingdom host shifts between closely related parasite species: between angiosperms and oomycetes, and from diatoms/brown algae to angiosperms. Switching between such phylogenetically distant hosts is generally unknown in host-dependent eukaryote parasites. We reveal novel plasmodiophorid lineages in soils, suggesting a much higher diversity than previously known, and also present the most comprehensive phytomyxid phylogeny to date.

**Conclusion:**

Such large-scale host shifts between closely related obligate biotrophic eukaryote parasites is to our knowledge unique to phytomyxids. Phytomyxids may readily adapt to a wide diversity of new hosts because they have retained the ability to covertly infect alternative hosts. A high cryptic diversity and ubiquitous distribution in agricultural and natural habitats implies that in a changing environment phytomyxids could threaten the productivity of key species in marine and terrestrial environments alike via host shift speciation.

## Background

Phytomyxea (phytomyxids) are a poorly known group of obligate biotrophic, endobiotic parasites of plants, diatoms, brown algae, and oomycetes [1,2]. Their phylogenetic position was long debated but molecular phylogenies now robustly place them within the eukaryote supergroup Rhizaria, as sister group to the omnivorous vampyrellid amoebae [3,4]. The group is subdivided into two orders: Phagomyxida (phagomyxids) and Plasmodiophorida (plasmodiophorids; [5,6]). Phagomyxids are very poorly known with only four characterised lineages, comprising two pairs of closely related species in the genera *Phagomyxa* (parasites of marine diatoms; [7]) and *Maullinia* (parasites of brown algae; [6,8]). In addition to these described species, several morphologically uncharacterised DNA-lineages have been detected by environmental sequencing [9,10].

Plasmodiophorids are better known because they include plant parasites causing significant diseases of crops including brassicas, potatoes, and grain crops (e.g. maize, rice, wheat, sorghum). The best studied species is the clubroot-causing *Plasmodiophora brassicae,* a parasite of crucifers which accounts for up to 10% loss of the worldwide production of *Brassica* crops [11]. Other well-studied species include *Spongospora subterranea,* which causes powdery scab of potato and can serve as a vector for Potato Mop Top Virus [12]. The otherwise symptomless *Polymyxa graminis* transmits economically important viruses to a number of grain crops [13] while *Polymyxa betae* is the vector for beet necrotic yellow vein virus, the cause of sugar beet “rhizomania” [14]. Given the impact of plasmodiophorids on staple crops it is not surprising that research has been focussed on the species causing economic damage, while research on species from outside of agricultural environments has been rare.

Phytomyxids are biotrophic parasites, i.e. they infect and multiply in a host without killing it, as opposed to hemi-biotrophic or nectrotrophic parasites, which ultimately kill the host [15]. Upon infection phytomyxids use an arsenal of methods to manipulate host physiology and to escape defence responses [16,17]. A dependence on living hosts to survive and to complete their life cycle is invariably associated with high host specificity because of the highly specialised interactions involved [16,17].

Phytomyxids have a complex life cycle consisting of two functionally different zoosporic stages (primary and secondary), which upon infection of a suitable host develop into multinucleate plasmodia inside the infected host cell (for a detailed review of the life cycle see e.g. [18]). These plasmodia either develop into a zoosporangium which rapidly releases large numbers of secondary zoospores or into resting spores which are the survival stage of the parasite and from which primary zoospores are released [1,18,19]. The resting spores can be aggregated into so-called sporosori and this arrangement is characteristic for each genus. Because of their complex six-stage life cycle [1,18], their small size (3-6 μm; [20]), and the fact that some life cycle stages are difficult to find and identify morphologically, there have been no recent studies of plasmodiophorid biodiversity; previous studies date back to the pre-molecular era [1,20,21]. Phytomyxids are not well represented in DNA databases: sequences are only available for nine of the 41 described species. An ecologically and evolutionary important group missing from molecular phylogenies are the phytomyxean oomycete parasites. These were originally placed in the now superseded taxon Plasmodiophoromycetes, then still included within Fungi [22]. The taxonomy of one genus of oomycete parasites–*Woronina* spp.–was studied two decades ago using ultrastructural studies, but *Woronina* has not previously been included in molecular phylogenies including more recently discovered parasites of other stramenopile hosts [7,8].

Molecular biodiversity studies are important for understanding the evolution, abundance and the possible economic and ecological impact of parasites, especially as global parasite numbers are thought to be hugely underestimated [23]. This unknown parasite biodiversity may have a large impact in a changing environment, where the emergence of existing and ‘novel’ diseases can have devastating effects [23-25]. Therefore, a targeted study of phytomyxid biodiversity is overdue, because of their known potential to damage crops directly by causing diseases or indirectly by transmitting viruses [2,15].

To further investigate phytomyxid biodiversity we used a parallel approach to screen selected soil ecosystems: microscopically examining potential hosts, coupled with targeted molecular screening of rhizosphere-associated and root-free soil from the same sites. Soil samples were obtained from sites ranging from intensively farmed to those with restricted anthropogenic input; we hypothesised that sites harbouring different host communities will also differ in parasite biodiversity. We also aimed to test whether phytomyxids that parasitise oomycetes group with phagomyxids or plasmodiophorids using a combination of isolation, microscopic identification, and 18S rDNA clone libraries. Through analysing 18S rDNA sequences we identified cross kingdom host shifts within Plasmodiophorida and Phagomyxida.

## Results

### New phytomyxid isolates and phylogenetic analyses

**Figure 1:**
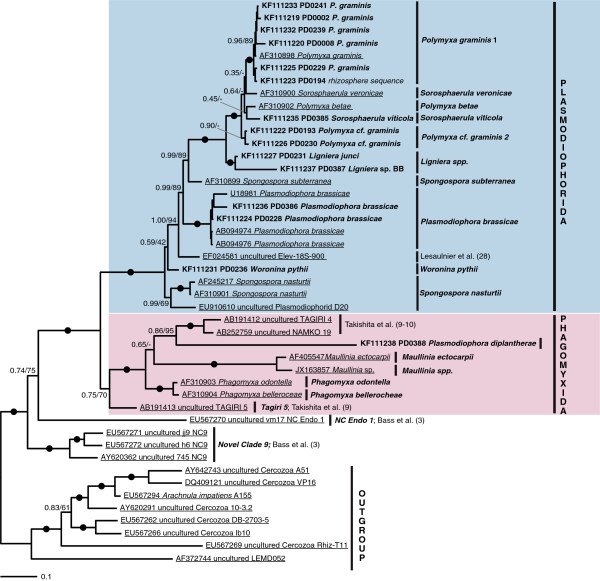
**Bayesian analysis of 18SrDNA sequences generated from isolates (bold) plus a comprehensive representation of Phytomyxea sequences from GenBank (underlined).** 44 sequences, 1795 positions. Bayesian support values shown if 0.70 or higher, or applied to a bipartition of particular interest. RAxML bootstraps shown for values of 50% and higher. Nodes with a support higher than 0.95/95 are marked by black circles. Further information to the isolates sequenced can be found in Additional file 1: Table S1.

**Figure 2:**
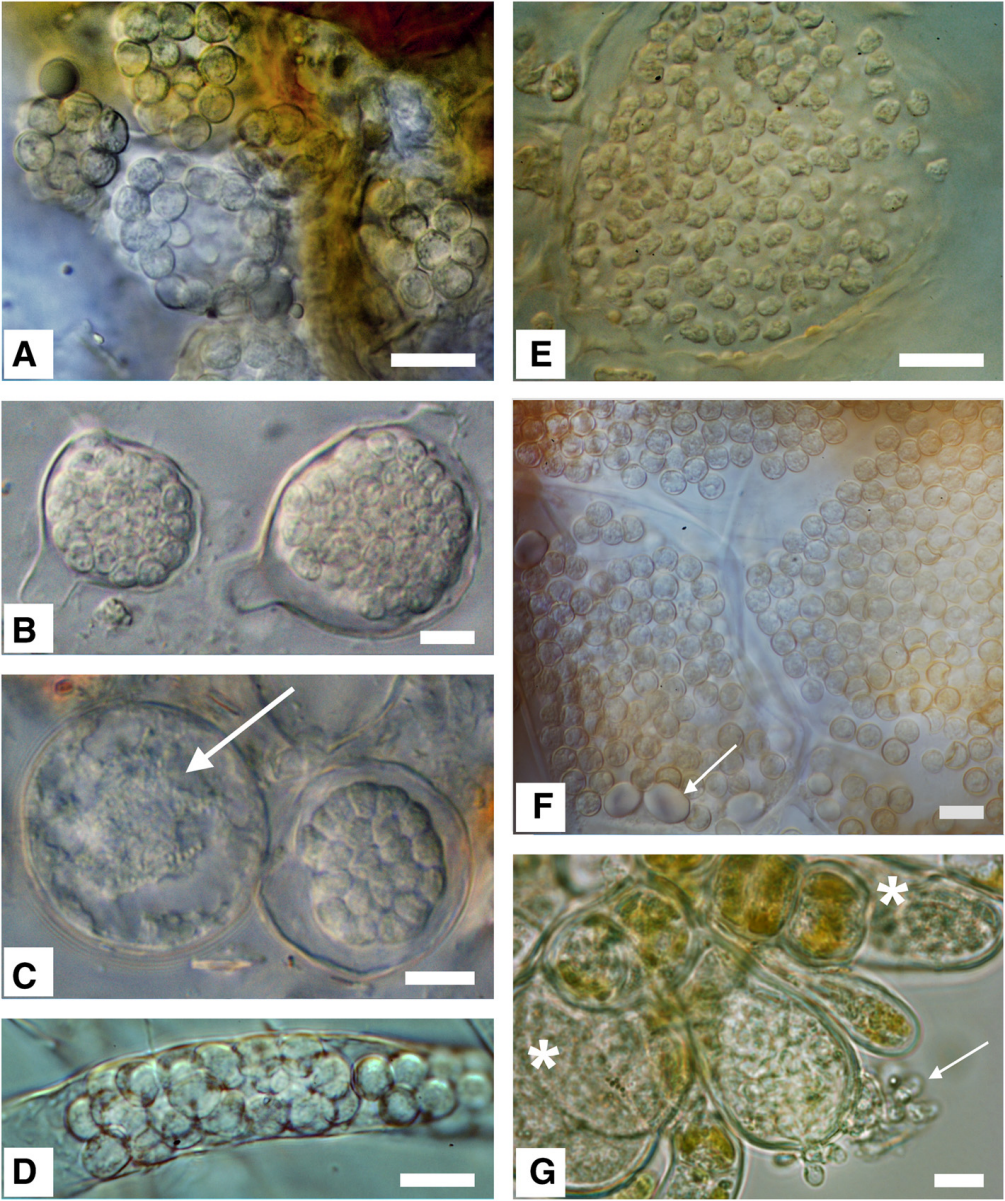
**Morphology of resting spores from selected phytomyxids.** Bar?=?10 μm: left column: Plasmodiophorida, right column: Phagomyxida. Left top to bottom: **2A**: *Sorosphaerula viticola*: hollow sporosori in the roots of *Vitis* sp. **2B**: *Woronina pythii*: resting spores in *Pythium* sp. **2C**: *W. pythii* in *Pythium sp.*: lobose plasmodium, just starting to develop into resting spores (arrow); right more or less mature resting spores. **2D**: *Ligniera junci*: resting spores in the root hairs of *Juncus effusus*. Right top to bottom: **2E**: *Maullinia* sp. resting spores in *Durvillea antarctica*. The resting spores are slightly irregular in size and shape. **2F**: *Plasmodiophora diplantherae*: Resting spores in *Halodule* sp. Resting spores are in enlarged cells of the host, Arrow: starch grains. **2G**: *Maullinia ectocarpii*: hatching zoospores (arrow) from an enlarged infected cell of the host *Ectocarpus fasciculatus*. Asterisk: plasmodia in enlarged host cells.

18S rDNA sequences were generated for 20 phytomyxid isolates for which resting spores could be characterised by microscopy (Figure 1, Additional file 1: Table S1). These isolates were identified following morphotaxonomic concepts (Figure 2; [20]). Resting spore size, shape, and arrangement are the primary defining taxonomic characters of phytomyxids, along with the identity of their host species [1,26]. The new sequences were included in Bayesian and Maximum Likelihood phylogenetic analyses with phytomyxid sequences from previously characterised specimens, phylogenetically informative environmental sequences [9,10,27], and the closely related environmental lineage ‘Novel Clade 9’ [3]. Our new isolates represent six previously described species as well as two undescribed lineages (*Maullinia sp.,* Figure 2e; *Polymyxa* cf. *graminis* 2; Additional file 1: Table S1). We provide the first 18S rDNA sequences for *Sorosphaerula viticola* (four isolates from the vine *Vitis* spp*.,* Figure 2a), *Ligniera junci* (two isolates from the rush *Juncus* spp., Figure 2d), *Woronina pythii* from the oomycete *Pythium* sp. (Figure 2b, c), and *Plasmodiophora diplantherae* from the angiosperm sea grass *Halodule wrightii* (Figure 2f). We also sequenced five isolates of *Polymyxa graminis* from the grass *Poa* sp*.*, two of *Plasmodiophora brassicae* from the crucifers *Sinapis* sp. and *Brassica* sp., and a plasmodiophorid we identified as *P. graminis* from *Poa* sp. (referred to as *Polymyxa* cf. *graminis* 2) which formed a distinct clade branching basally to *P. graminis, S. veronicae, P. betae,* and *S. viticola* in the 18S phylogeny*.* The two orders Phagomyxida and Plasmodiophorida, as previously conceived, were robustly supported. Seven distinct lineages could be resolved within the Phagomyxida and eleven within the Plasmodiophorida. According to this analysis, all plasmodiophorid genera (as currently circumscribed) that are represented in our phylogeny by more than one species are polyphyletic (Figure 1).

### Cross-kingdom host shifts of closely related Phytomyxids

**Figure 3:**
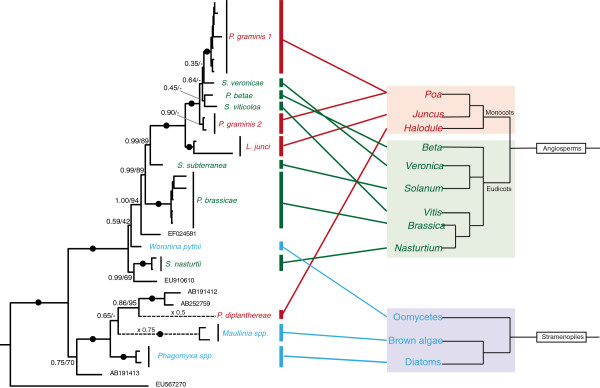
**Diagrammatic co-phylogeny comparing phytomyxid taxonomy and phylogenetic relationship of their hosts.** Phytomyxea are connected to their primary host species to highlight the degree of host switches between and within vascular plant and stramenopile hosts. Left hand side: a modified version of the tree shown in Figure 1. Dotted branches were shortened (scale indicated). Right hand side: diagrammatic tree of green plants and stramenopiles (based on [4,28,29]). Red?=?monocot plant host, Green?=?eudicots plant hosts; Blue?=?stramenopile host.

We show that plasmodiophorids infecting angiosperms are paraphyletic with respect to those infecting oomycetes, as are stramenopile-associated Phagomyxida to the angiosperm parasite *Plasmodiophora diplantherae. P. diplantherae* branches robustly within phagomyxids, sister to two environmental sequences which were found in clone libraries of deep-sea/anoxic saline sediments [9,10]. The oomycete parasite *Woronina pythii* grouped within plasmodiophorids, closely related to *Spongospora nasturtii* and *Plasmodiophora brassicae*, both parasites of cruciferous plants (Figure 3). However, the branching order of these lineages within Plasmodiophorida is not resolved; *Woronina* may in fact be the most basally branching plasmodiophorid lineage. Light microscopy of *W. pythii* showed that size, arrangement and development of the resting spores from sporogenic plasmodia were very similar to that seen in other plasmodiophorids (e.g. *P. brassicae*, *S. veronicae*, *S. viticola*) (Figure 2B, C). The resting spores of *P. diplantherae* (Figure 2F) on the other hand strongly resembled those of the phagomyxid *Maullinia* sp. (Figure 2E) in ornamentation and a loose aggregation at maturity.

**Figure 4:**
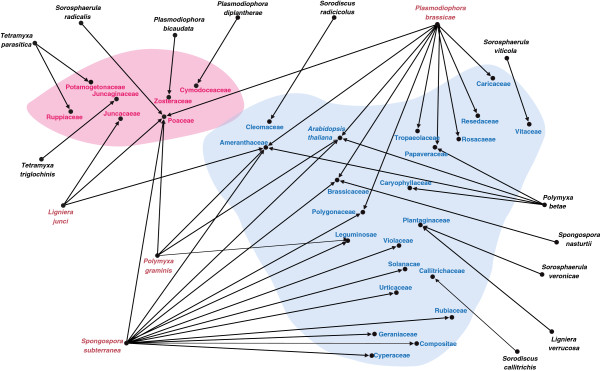
**Network analysis of plant parasitic Phytomyxea and potential host plants (shown as plant families following the taxonomy suggested on** http://www.theplantlist.org **, 20.06.2013).** Phytomyxids can use alternative host plants during their life cycle. for the majority of species only haphazard reports of the host range are available, while for some agriculturally important species like of *Spongospora subterranea, Polymyxa graminis*, *P. betae,* and *Plasmodiophora brassicae* extensive host range studies are available, leading to a seemingly wider host range of these species. It is noteworthy that, despite this bias, all species for which targeted host range studies exist hosts belonging to monocot and dicot host plants can be found. Pink?=?monocot plant families, Blue?=?dicot plant families, Red font?=?Phytomyxids for which hosts belonging to both the monocot and dicot hosts were described.

Furthermore, plasmodiophorids are known to rarely infect a range of so-called alternative hosts in which very limited (i.e. to single host cells) infections can be observed. Therefore, we conducted a comprehensive literature study to identify and summarise possible alternative hosts (Figure 4, Additional file 1: Table S2). Interactions with alternative hosts remain highly understudied; however one characteristic common to those known is that resting spores are rarely or never formed. According to these data some plasmodiophorid species can infect host plants belonging to two or more plant families; *Ligniera junci*, *Polymyxa graminis*, *Plasmodiophora brassicae,* and *Spongospora subterranea* are known to parasitize both monocot and eudicot host species (Figure 4). Plasmodiophorid parasites of oomycetes and the phagomyxid *M. ectocarpii* (Figure 2G) can infect a range of distantly related oomycetes or brown algae respectively (Additional file 1: Table S2).

Despite an extensive sampling of living plants in this study, no alternative host associations in nature could be confirmed by microscopic observation. However, direct and clone sequencing of rhizosphere samples revealed phytomyxid sequences associated with possible alternative host plants (Additional file 1: Table S3). Sequences closely related to *Polymyxa graminis* 1 (primary hosts Poaceae) were found associated with *Taraxacum* sp., *Veronica* sp., and *Vitis* sp.; sequences with a 96.4-97.2% similarity to *Polymyxa betae* (primary hosts Amaranthaceae) with *Vitis* sp.; a *Spongospora nasturtii*-like sequence (96.1% similarity to AF245217; primary host *Nasturtium* spp., Brassicacae) from *Carex* sp.; and *S. subterranea*-like sequences (98.4% similarity, primary hosts Solanaceae) from *Trifolium* sp. The nature of these associations remains unclear; microscopy of the associated root samples was not conclusive and sequence data alone does not provide sufficient information about organismal associations.

The division of Phytomyxea into Phagomyxida and Plasmodiophorida was confirmed by our phylogenetic (Figure 1) and morphological analyses, and also corresponded to the environment in which the host species were found rather than host taxonomy: plasmodiophorids are a monophyletic lineage of parasites of land plants and soil-dwelling oomycetes, while phagomyxids are a monophyletic lineage infecting marine plants and stramenopiles. We tested our plasmodiophorid-specific primer combination on phagomyxid isolates (*Maullinia* sp., *Maullina ectocarpii*, *P. diplantherae*), and on a selection of marine algae (brown, red, green) and marine (littoral) sediment from the South coast of the UK (n?=?38). All results were negative: no plasmodiophorids were detected in marine environments.

### Environmental sequence analysis of plasmodiophorids

**Figure 5:**
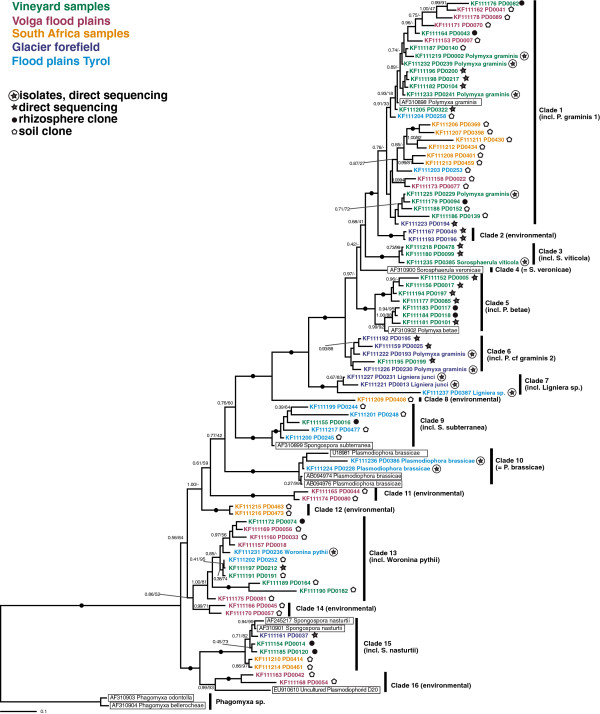
**Bayesian analysis of environmental 18SrDNA sequences generated in this study plus a representative selection of Phytomyxea sequences from GenBank (highlighted by boxes).** 94 sequences, 1690 positions. Bayesian support values/RAxML bootstraps are shown if 0.70/50% or higher, or applied to a bipartition of particular interest. Nodes with a support higher than 0.95/95 are marked by black circles. Sequences are colour coded according to the origin of the sample, and the type of sample (soil sample, rhizosphere, isolate) are indicated by icons.

Plasmodiophorid-specific PCRs were carried out on 385 individual rhizosphere and soil samples (Additional file 1: Table S4), of which 153 gave positive results. After pooling of amplicons for cloning, a total of 303 18S rDNA reads were obtained from 21 clone libraries and direct sequencing of rhizosphere samples (n?=?19). After quality and chimera checks 295 of these sequences were analysed further. A total of 81 different plasmodiophorid sequence-types (18S-types, defined as described in the Methods) were detected (Additional file 1: Table S1, Additional file 1: Table S3). These grouped into 16 phylogenetically well supported clades within the Plasmodiophorida (Figure 5). Ten clades included sequences from at least one microscopically confirmed isolate, whereas six comprised only environmental 18S-types. Clade 4 (*S. veronicae*) and Clade 8 (environmental) are monotypic clades. The environmental lineages Clade 2, Clade 11, Clade 12, Clade 14 each consist of two 18S-types. Clade 3 (incl. *S. viticola*), Clade 7 (incl. *L. junci*) and Clade 16 (environmental) comprise three 18S-types each, while Clade 6 (incl. *P. cf. graminis 2*) consists of four 18S-types. Clade 10 (all *P. brassicae*) contains five, Clade 9 (incl. *S. subterranea*) six, Clade 15 (incl. *S. nasturtii*) seven and Clade 5 (incl. *P. betae*) eight, respectively. Clade 13 which contains *W. pythii* was shown to comprise eleven distinct 18S-types from five independent samples, indicating a substantial diversity of probable oomycete parasites. Clade 1 (incl. *P. graminis* 1) includes thirty 18S-types of which five belong to isolates.

All six unidentified environmental lineages (Clades 2, 8, 11, 12, 14, 16) were each found in one geographically/ecologically distinct sample set only: Clade 2 was found associated with plant roots of a glacial succession site, Clade 8 (monotypic) and Clade 12 were found only in South Africa fynbos soil samples, and Clade 11, Clade 14 and Clade 16 in samples from Volga flood plains.

Positive PCRs from rhizosphere samples generally could be directly sequenced without cloning, while soil samples needed to be cloned even if the diversity within the individual samples was generally low (Additional file 1: Table S4). Every soil sample contained at least two 18S-types (Additional file 1: Table S4). Nevertheless the sequenced plasmodiophorid 18S-types detected in any individual clone library were never assigned to more than four of the 16 clades defined in Figure 5.

From a German vineyard sampled repeatedly over a period of two years only five 18S-types (Figure 5, Additional file 1: Table S4) could be identified from 78 sequence reads obtained from four independent soil clone libraries. At the same time, three different species of plasmodiophorids were morphologically identified in plant samples from the same site: *Sorosphaerula viticola* was abundant in *Vitis* sp. roots, *P. betae* was identified from the roots of a *Chenopodium* sp., and *Polymyxa graminis* was frequently seen in the roots of *Poa* species. The latter was the most abundant sequence type from the soil clones and rhizosphere samples, while *S. viticola* and *P. betae* were not detected in the soil clone libraries, but were found when rhizosphere samples from primary host plants were sequenced. Therefore the 18S-types detected morphologically were a subset of those detected by sequencing: all species identified by microscopy were detected in the respective soil/rhizosphere/plant samples at least once, but not all DNA types could be assigned to actual isolates. Across all samples, plasmodiophorids were generally more easily detected in the rhizosphere of their assumed host than in neighbouring bulk soil.

## Discussion

Two host shifts between plant and stramenopile hosts were found in the phytomyxids (Figures 1 and 3). Prior to this study it was thought that Phagomyxida were exclusively parasites of stramenopiles and Plasmodiophorida exclusively plant parasites [5]. However, we have shown that *Woronina pythii,* a parasite of the oomycete *Pythium spp.*, branches within the Plasmodiophorida (Figure 1). Conversely, *Plasmodiophora diplantherae,* a parasite of an angiosperm sea grass, branches robustly amongst parasites of diatoms and brown algae. There is to our knowledge no other group of obligate biotrophic eukaryote parasites showing such pronounced cross-kingdom host shifts. In the eukaryotes cross-kingdom host shifts are known from less host-dependent opportunistic or necrotrophic parasites belonging to fungi and oomycetes [28,30], while host shifts within other biotrophic parasites seem to occur only between relatively closely related hosts [31-34]. Biotrophic oomycetes include parasites of both nematodes (*Haptoglossa* spp.) and brown algae (*Eurychasma* spp.), but these parasites show distinct morphologies and adaptations to their hosts [28] suggesting strongly divergent adaptations to different host conditions. Phytomyxid parasites on the other hand have a very similar lifecycle, morphology and development inside the host (Figure 2) without any obvious specialised adaptation to a particular group of hosts. Thus, in phytomyxids, closely related lineages can be parasites of a wide range of fundamentally different hosts (autotrophic or heterotrophic) in different environments (marine, soil, freshwater).

Our morphological analyses are concordant with the cross-kingdom host jumps as revealed by molecular phylogenies. Resting spores and development of *W. pythii* strongly resemble that of plasmodiophorid parasites of vascular plants, especially *Spongospora* spp. and *Sorosphaerula* spp. (Figure 2a-d). The resting spores of *P. diplantherae* are morphologically very similar to those recently identified in *Maullinia* sp. from the kelp *Durviellea antarctica* in their ornamentation and loose aggregation (Figure 2e-g). Such loose arrangement of the resting spores is also seen in the plasmodiophorid *P. brassicae*, but ultrastructurally *P. diplantherae* is significantly different from *P. brassicae* (e.g. larger nucleus in *P. diplantherae* and differing numbers and arrangement of the synaptomal complexes) and all other plasmodiophorids [35]. Ultrastructural studies have shown that the timing and morphology of meiosis in *Woronina phythii* are very similar to those in the closely related *P. brassicae* (Figure 1, [22]). Taken together, our new sequence data, phylogenetic analyses, and complementary morphological investigations question existing concepts of phytomyxid parasitism. Based on our phylogenetic analysis (Figure 1) we infer that in the marine environment parasitism of stramenopiles developed first, followed by a host switch towards angiosperm hosts. The direction of the host switch within plasmodiophorids is equivocal, because of the unresolved branching order of the deep branching taxa, including *Woronina*. However, we raise the hypothesis that the ancestral plasmodiophorid might have been a parasite of stramenopiles, which then bifurcated into lineages with plant hosts and those with oomycete hosts. Further sampling of the lineage diversity at the base of the phytomyxid radiation and determination of their parasitic status would enable this hypothesis to be tested further, as would characterisation of Novel Clade 9, the immediate sister group to phytomyxids [3], and multi-gene phylogenomic analyses of characterised members of the whole vampyrellid-phytomyxid radiation.

During the last decade it has become clear that cases of co-speciation between parasites and their hosts are rare and that the diversification of obligate parasites is more often shaped by host shifts [36]. However, how parasites and their hosts co-evolve alongside each other remains a key issue in understanding both partners [36,37], and can also influence policy decisions on quarantine and control measures by authorities [38]. Phytomyxids require their primary host plant to complete their life cycle and to successfully reproduce and persist in the environment, but they can also develop infections at a low level in many alternative, non-primary host plants Figure 4, Additional file 1: Table S1; [39-41]. However, the nature of such interactions remains enigmatic. We detected phytomyxid sequences in the rhizosphere of potential alternative host plants, but could not microscopically confirm any infections of alternative hosts. The ability to infect diverse hosts without causing a distinct, visible response or by causing restricted infections over a number of generations could be a highly advantageous trait when the primary host is transient or rare. Restricted infections of phytomyxids in alternative hosts breach the first barrier to extending host range and may open the way to integrated adaptation to new hosts [42,43]. Based on our findings, we hypothesise that the main evolutionary driver of phytomyxids is related to their ability to infect a wide range of hosts, while the main constraint is ecological. We also conclude that speciation in phytomyxids is based on host shifts rather than other co-evolutionary processes [44,45] because of the fundamental (taxonomic) differences between the respective host species.

Phagomyxida are apparently exclusively marine and Plasmodiophorida are apparently exclusively non-marine, but we show that potentially the host range of both groups is larger than previously thought. A division in marine and freshwater/soil lineages is common in protists; very few free-living protist 18S-types are found in both marine and non-marine habitats [46], and in some protist groups evolutionary transitions from one environment to the other appear to be infrequent [47-49]. Ecological structuring also operates at taxonomically lower levels within plasmodiophorids: we revealed distinct, (novel) lineages and radiations from geographically and ecologically separated samples (South Africa, Volga, alpine glacial succession site, Figure 5). This supports the hypothesis that ecological barriers phylogenetically compartmentalise the diversification of phytomyxids.

Overall, we show a high 18S-type diversity of plasmodiophorids in soil ecosystems (Figure 5). Isolates from *P. graminis*, for example, seem to be highly variable at this level, e.g. average similarities 96.9?±?1.5% between the 30 individual 18S-types of Clade 1 (Figure 1). However, within this clade we could identify no pattern in 18S-type–host-plant species association, although the branching order is not always well resolved. It is possible that this cryptic diversity displays the natural variation within *P. graminis*, as result of evolutionary failure to speciate along with their hosts [44]. But on the other hand it is possible that sequence clusters deriving from within similar environments in this clade represent *P. graminis* strains (or cryptic species) with different, locally advantageous host preferences, a phenomenon reported previously for *P. gramins*[50,51], but otherwise are currently unknown. The nature of species complexes or cryptic species which are specialised on specific hosts/environments is increasingly recognised for fungal and oomycete plant pathogens [52,53] and is likely to be valid for phytomyxids in general and *P. graminis* in particular [50,51]. Cryptic speciation and infection of novel hosts via host shift speciation is considered to be the most frequent reason for the development of emerging diseases in fungi and oomycetes [42,53,54]. Host shifts between closely related parasites can also lead to more severe disease symptoms [42]. Our findings raise the possibility that host shifts and subsequent adaptation of phytomyxids to new hosts could become more frequent or intensified in changing environments.

The overall diversity of phytomyxids in individual samples was relatively low, especially when compared to the diversity found within many groups of free-living protists, where often tens of relatively closely related but biologically distinct 18S-types can be found in a few cm^3^ of soil [55-58]. In soil samples of the intensively sampled vineyard (a total of 300 g soil DNA was extracted and analysed during the two year experiment) only five 18S-types belonging to two clades (clade 1, clade 13) could be identified via soil clone libraries while at the same site three species were identified microscopically and from targeted rhizosphere clones (*P. graminis–clade1*, *P. betae–clade 5*, *S. viticola–clade 3*). This contrasts with the detection of eleven 18S-types belonging to five species found in a smaller volume of targeted root and rhizosphere samples from the same site, highlighting that the soil clone libraries on their own underestimate the abundance and biodiversity of plasmodiophorids.

## Conclusion

Within each of the two major phytomyxid clades there have been cross-kingdom host shifts between closely related parasite taxa: from stramenopile to angiosperm hosts in phagomyxids and between the same two host groups by plasmodiophorids. Such large-scale host shifts between closely related obligate biotrophic eukaryote parasites is to our knowledge unique to phytomyxids. The fundamental constraint in lineage radiation seems to be the environment: plasmodiophorids and phagomyxids are monophyletic groups with, respectively, non-marine and marine hosts. Environment type also seems to compartmentalise lineage radiation within plasmodiophorids at lower taxonomic levels. Our results indicate that the current research focus on a few economically significant phytomyxid lineages may lead to a limited understanding of this highly varied and largely enigmatic group of parasites of plants and stramenopiles. Some phytomyxid plant pathogens also have the ability to develop at least part of their life cycle in alternative hosts. Further work into phytomyxid diversity and their interactions with hosts in marine and non-marine (e.g. freshwater) habitats will determine (i) if high level taxonomic host shifts are more frequent, (ii) whether other high level eukaryote taxa (phyla, supergroups) are used as hosts, and (iii) whether the diversity and spatial occurrence of more closely related (i.e. around species-level) phytomyxid lineages is most strongly determined by host specificity or a wider range of environmental factors.

## Methods

### Sample collection

Samples were collected from a variety of environments (Additional file 1: Table S5), including 40 individual soil samples and 10 plant samples collected repeatedly in 2009/2010 from a German vineyard (Bernkastel, Moselle valley). The soil samples were analysed individually, whereas the plant samples (blocks of 30?×?30 cm) were pooled into plant families prior further processing to keep the sample size manageable to allow a parallel DNA-based and microscopic study. Potential host plants for phytomyxids were collected independently since 2004 to increase the taxon sampling for phylogenies. Plant samples were inspected microscopically, and if a plasmodiophorid parasite was found, DNA was extracted and sequenced (Additional file 1: Table S1).

### DNA extraction and PCR

Soil samples were sieved through a 2 mm mesh size metal sieve (sterilised by flaming with Ethanol between samples) to remove stones and other large debris before DNA extraction. Root samples were picked directly from sample blocks using sterile tweezers, carefully rinsed with sterile distilled water, and used for DNA extraction. This approach of root sampling allowed tracing the selected root to the respective plant also reduced the risk of washing away fine roots which would result in a considerable loss of root biomass available for analyses.

Each soil sample was extracted in three replicates (0.5 g each). A fourth replicate of each soil sample was spiked with +/- 0.5 cm^2^ fungal mycelium as positive control (*Roesleria subterranea* strain *IB 2005/0506*), ensuring that negative results are not false negatives caused by PCR inhibitors. A negative control and a positive control without soil (*R. subterranea* strain *IB 2005/0506*) were included in every extraction.

Soil DNA and DNA from plant roots was extracted as described [59] or using the MoBio UltraClean Soil DNA isolation kit (South Africa samples). Each DNA extract was tested individually using fungal primers (V9G, LS266) [60] which served as yet another quality control for the DNA extraction to exclude false negative results because fungi are present in all samples, hence all samples should amplify with fungal primers.

To detect plasmodiophorids a nested PCR using two sets of 18S rDNA primers was used. First round PCR Primers were Plas1f (5′-TCAgTgAATCTgCggATggC-3′) and Plas 1r (5′-ggTgCSKCKAgRTVCAAgAggC-3′); second round primers were Plas2f (5′-TggATgTACgAGAgTACTACATgg-3′) and Plas2r (5′-CgTTgAACCTAgCATTgTAgCg-3′).

PCR-reactions (20 μL) contained final concentrations of 0.2 mM dNTPs, 0.1 μM of each primer, 1?×?PCR-reaction-buffer, 2.5 mM MgCl_2_, 2 μg mL^-1^ Bovine Serum Albumin, 0.5 U of Taq-Polymerase (Dream Taq, Fermentas) and 3 μl DNA. A touchdown PCR- protocol was used: 96°C for 4 min initial denaturation, followed by two cycles of 96°C for 25 s, 65°C for 25 s and 72°C for 1.5 min succeeded by two cycles each with an annealing temperature of 60°C and 58°C and finally 30 cycles with an annealing temperature of 54°C and completed by a final elongation step at 72°C for 10 min.

PCR products were cloned using the Fermentas insT/A cloning Kit (Thermo scientific, Germany). Positive clones were identified using the PCR primers described above. PCR products were PEG-purified (http://www.mcdb.lsa.umich.edu/labs/olsen/files/PCR.pdf) and sequenced using the specific primers (Macrogen, Korea). Sequences used in this study were deposited in Genbank accession numbers KF111152-KF111238.

### Sequence manipulation, chimera check, and sequence selection

Sequences were initially blasted against GenBank (blastn) to identify phytomyxid sequences. A primary alignment of all sequences was created using MAFFT [61] implemented in Geneious pro (version 5.6.4; [62]) and this alignment was improved manually using BioEdit (version 7.0.5.3 [63]). To exclude chimeras Bellerophon [64] was used. We also compared trees computed from the alignment divided into half, and compared the respective positions of all sequences on these trees. Sequences which showed significantly different branching orders on the two trees were excluded. To group similar and/or identical sequences PhyML [65] and MrBayes [66] trees were calculated and highly similar sequences grouped and subsequently removed: sequences from individual clone libraries with a similarity of 98% or higher were treated as belonging to a single 18S-type; only one representative sequence of each 18S-type was used for subsequent analyses. The cut off-value of 98% sequence similarity was not applied to sequences from isolates or for sequences from independent clone libraries, as these sequences were considered to be ecologically and phylogenetically informative.

### Phylogenetic analyses

For phylogenetic analysis sequences were re-aligned using the l-ins-i algorithm in MAFFT and refined by eye in MacGDE. The refined alignment was analysed in RAxML BlackBox (GTR model with CAT approximation (all parameters estimated from the data); bootstrap values were mapped onto the tree with the highest likelihood value. Bayesian consensus trees were also constructed using MrBayes v 3.1.2 in parallel mode. Two separate MC3 runs with randomly generated starting trees were carried out for 6 M generations each with one cold and three heated chains. The evolutionary model included a GTR substitution matrix, a four-category autocorrelated gamma correction and the covarion model. All parameters were estimated from the data. Trees were sampled every 100 generations. 2 M generations were discarded as “burn-in” (trees sampled before the likelihood plots reached a plateau) and consensus trees constructed from the returning sample.

### Light microscopy

Plant roots were carefully washed with tap water to remove soil and organic matter attached to the roots. Samples were transferred into petri dishes with sterile, distilled water and roots that were still part of the plant of interest were selected using forceps. These roots where then either used directly for microscopy (in water) or stained with cotton blue. The roots were screened using 250 × magnification and DIC on a Nikon Optiphot light microscope, and phytomyxean structures confirmed using a 1250 × magnification.

### Bait tests for the isolation of oomycete parasitic phytomyxids

A few grains of soil were added to a 9 cm petri dish filled with a mixture of sterile tap water and distilled water (1:1). Hemp seed (3 seeds per 9 cm petri dish), niger seed (*Guizotia abyssinica*, 3-5 seeds per 9 cm petri dish) and sesame seeds (3-5 seeds per 9 cm petri dish) were used as bait respectively. From Lohbach, Rotmoos and Bernkastel soils 20 parallel samples were tested with each bait. Samples were incubated at 10°C in the dark and checked for oomycete growth once a week for one month. The low incubation temperature slowed the growth of zygomycetes (esp. *Mucor* spp. and *Mortierella* spp.), hyphomycetes (esp. *Trichoderma* spp., *Fusarium* spp.) and bacteria which out-competed the oomycetes quickly when incubated at 20°C. Baiting cultures were first screened for oomycete growth using a reflected light microscope with 250?×?magnification. Samples which contained oomycetes were screened in an inverted microscope for the presence of cytosori. If resting spores could be identified, a microscope slide was prepared and the presence of the parasite confirmed using a Nikon Optiphot light microscope (DIC, 100?×?oil). Samples with confirmed infections were frozen for subsequent DNA extraction and analyses.

### Host range meta-study

Potential primary and secondary hosts of phytomyxids were determined [20] supplemented by more recent studies on the host range of *S. subterranea*[41,67], *Polymyxa graminis*[39,50,68], *Polymyxa betae*[69,70] and *Plasmodiophora brassicae*[40]. Host plants were pooled into plant families (Additional file 1: Table S2) and host–phytomyxid associations collated in an edges table (using directed, but un-weighted edges) suitable for import into Gephi 0.8.1 beta (https://gephi.org/). Nodes were defined by default from the edges table in the data laboratory module, and the initial network graph was created using the “Force Atlas” layout (settings differing from default: repulsion strength?=?10000; auto stabilisation strength?=?100; gravity?=?100). The resulting network was manually modified in Gephi until the final layout shown in Figure 4 was obtained. Fine adjustments to the layout (e.g. font colours, background colours) were made using Adobe Illustrator.

## Availability of supporting data

Sequences used in this study were deposited in Genbank accession numbers KF111152-KF111238. Any associated data are summarised in Additional file 1.

## Competing interests

The authors declare that they have no competing interests.

## Authors’ contributions

SN and MK designed research, SN performed research, SN, MK, SB and DB analyzed data and wrote the paper. All authors read and approved the final manuscript.

## Supplementary Material

Additional file 1: Table S1 Microscopically observed isolates. **Table S2.** Data and references used for meta-analysis of phytomyxid host range, including data used to compile Figure 4. **Table S3.** Dataset of environmental sequences shown in Figure 5, including information on samples and abundances of the individual 18-S types in the clone libraries. **Table S4.** Sample numbers, positive detections using the plasmodiophorid specific primer strategy, and 18S rDNA types within each sample pool. **Table S5.** Samples, sampling dates and sampling sites. Click here for file
