# Supplementary material for: Cross-kingdom host shifts of phytomyxid parasites
Source: BMC Evol Biol. 2014 Feb 23;14:33. doi: 10.1186/1471-2148-14-33 (PMC4016497; doi:10.1186/1471-2148-14-33)
Supplement: Additional file 1: Table S1 — Microscopically observed isolates. Table S2. Data and references used for meta-analysis of phytomyxid host range, including data used to compile Figure 4. Table S3. Dataset of environmental sequences shown in Figure 5, including information on samples and abundances of the individual 18-S types in the clone libraries. Table S4. Sample numbers, positive detections using the plasmodiophorid specific primer strategy, and 18S rDNA types within each sample pool. Table S5. Samples, sampling dates and sampling sites. [file 1471-2148-14-33-S1.pdf]

Supplementary Material

Cross-kingdom host shifts of phytomyxid parasites

Neuhauser Sigrid, Kirchmair Martin, Bulman Simon, Bass David

Supplementary Tab S1: Microscopically observed isolates.

Supplementary Tab S2: Data and references used for meta-analysis of phytomyxid host range, including data used to compile Supplementary Fig. S2.

Supplementary Tab S3: Dataset of environmental sequences shown in Fig. 3, including information on samples and abundances of the individual 18-S types in the clone libraries.

Supplementary Tab S4: Sample numbers, positive detections using the plasmodiophorid specific primer strategy, and 18S rDNA types within each sample pool.

Supplementary Tab S5: Samples, sampling dates and sampling sites.

References cited in Supplementary Material

Supplementary Tab. S1: Microscopically observed isolates, origin, host plant, including sample code and Genbank accession numbers. Taxonomic assignment of the species follows Fig. 1.

Phytomyxid	Host plant	Sampled	Sample code	accession number
Ligniera junci	*Jucus triglumis*	Rotmoostal, Ötztal, Austria	PD0013	KF111221
Ligniera sp.	*Poa sp.*	Wet meadow, Brandenberg, Austria	PD0387	KF111237
Ligniera junci	*Juncus effusus*	Münster, Austria	PD0231	KF111227
Maullinia sp.	*Durvillea antarctica*	Coliumo bay, Chile[1]	-	JX163857
Plasmodiophora brassicae	*Broccoli (Brassica oleracea)*	Axams, Tirol, Austria	only microscopy	-
Plasmodiopohra brassicae	*Sinapis arvensis*	Ampass, Tirol, Austria	PD0228	KF111224
Plasmodiophora brassicae	*Chinese cabbage (Brassica napa ssp. pekinensis)*	Kematen, Tirol, Austria	PD0386	KF111236
Plasmodiophora diplantherae	*Halodule wrightii*	Gulf of Mexico[2]	PD0388	KF111238
Polymyxa graminis	*Poa sp.*	Bernkastel, Germany	PD0002	KF111219
Polymyxa graminis	*Poa sp.*	Bernkastel, Germany	PD0008	KF111220
Polymyxa graminis	*Poa sp.*	Bernkastel, Germany	PD0229	KF111225
Polymyxa graminis	*Poa sp.*	Kiedrich, Germany	PD239	KF111232
Polymyxa graminis	*Poa sp.*	Wehlen, Germany	PD0241	KF111233
Polymyxa cf graminis 2	*Poa alpina*	Rotmoostal, Tirol, Austria	PD0193	KF111222
Polymyxa cf graminis 2	*Poa sp.*	Brandenberg, Austria	PD0230	KF111226
Sorosphaerula viticola	*Vitis sp.*	Kiedrich, Germany	PD0235 (=PD0385)	KF111230
Sorosphaerula viticola	*Vitis sp.*	Bernkastel, Germany	PD0385	KF111235
Sorosphaerula viticola	*Vitis sp.*	Bernkastel, Germany	PD0233 (=PD0385)	KF111228
Sorosphaerula viticola	*Vitis sp.*	Bernkastel, Germany	PD0234 (=PD0385)	KF111229
Woronina pythi	*Pythium sp.*	Lohbach, Tirol, Austria	PD0236	KF111231
Woronina pythi	*Pythium sp.*	Lohbach, Tirol, Austria	PD0332 (=PD0236)	KF111234

Supplementary Tab. S2: Meta analysis of phytomyxid host range, based on the Edges table imported to the Gephi software. Phytomyxids are polyphagic on diverse sets of vascular plant or stramenopile hosts. For plant pathogenic plasmodiophorids it is well established that a series of so called “alternative hosts” can be infected by *P. brassicae*, *P. graminis*, *P. betae* and *S. subterranea*. Host range studies for the oomycete parasite *W. pythii* and the brown algal parasite *M. ectocarpii* demonstrated a wide range of possible hosts. Plant taxonomy follows the suggestions of www.theplantlist.org and results as of 20.06.2013 were used. The table also collates information on a potentially wide host range of the oomycete and brown algal parasites, further supporting our hypothesis of the absence of a co-phylogentic signal in phytomyxids.

Source	Target	Target(2)	References
Ligniera_junci	Ameranthaceae	Eudicots	[3]
Ligniera_junci	Juncacaeae	Monocots	
Ligniera_junci	Poaceae	Monocots	
Ligniera_verrucosa	Plantaginaceae	Eudicots	[3]
Plasmodiophora_bicaudata	Zosteraceae	Monocots	[3]
Plasmodiophora_brassicae	Arabidopsis_thaliana	Eudicots	[3-4]
Plasmodiophora_brassicae	Brassicaceae	Eudicots	
Plasmodiophora_brassicae	Caricaceae	Eudicots	
Plasmodiophora_brassicae	Ameranthaceae	Eudicots	
Plasmodiophora_brassicae	Papaveraceae	Eudicots	
Plasmodiophora_brassicae	Polygonaceae	Eudicots	
Plasmodiophora_brassicae	Resedaceae	Eudicots	
Plasmodiophora_brassicae	Rosacaeae	Eudicots	
Plasmodiophora_brassicae	Tropaeolaceae	Eudicots	
Plasmodiophora_brassicae	Poaceae	Monocots	
Plasmodiophora_diplantherae	Cymodoceaceae	Monocots	[3]
Polymyxa_betae	Ameranthaceae	Eudicots	[3, 5-6]
Polymyxa_betae	Arabidopsis_thaliana	Eudicots	
Polymyxa_betae	Caryophyllaceae	Eudicots	
Polymyxa_betae	Ameranthaceae	Eudicots	
Polymyxa_betae	Papaveraceae	Eudicots	
Polymyxa_graminis	Arabidopsis_thaliana	Eudicots	[7-9]
Polymyxa_graminis	Ameranthaceae	Eudicots	
Polymyxa_graminis	Leguminoseae	Eudicots	
Polymyxa_graminis	Poaceae	Monocots	
Sorodiscus_callitrichis	Plantaginaceae	Eudicots	[3]
Sorodiscus_radicicolus	Cleomaceae	Eudicots	[3]
Sorosphaerula_radicalis	Poaceae	Monocots	[3]
Sorosphaerula_viticola	Vitaceae	Eudicots	[10]
Spongospora_nasturtii	Brassicaceae	Eudicots	[3]
Spongospora_subterranea	Ameranthaceae	Eudicots	[3, 11-12]
Spongospora_subterranea	Arabidopsis_thaliana	Eudicots	
Spongospora_subterranea	Compositae	Eudicots	
Spongospora_subterranea	Brassicaceae	Eudicots	
Spongospora_subterranea	Ameranthaceae	Eudicots	
Spongospora_subterranea	Cyperaceae	Eudicots	
Spongospora_subterranea	Leguminoseae	Eudicots	
Spongospora_subterranea	Geraniaceae	Eudicots	
Spongospora_subterranea	Poaceae	Eudicots	
Spongospora_subterranea	Polygonaceae	Eudicots	
Spongospora_subterranea	Rubiaceae	Eudicots	
Spongospora_subterranea	Solanacae	Eudicots	
Spongospora_subterranea	Urticaceae	Eudicots	
Spongospora_subterranea	Violaceae	Eudicots	
Tetramyxa_parasitica	Potamogetonaceae	Monocots	[3]
Tetramyxa_parasitica	Ruppiaceae	Monocots	
Tetramyxa_triglochinis	Juncaginaceae	Monocots	[3]
Sorodiscus_karlingii	Characeae	Charophyta	[3]
Tetramyxa_rhizophaga	Cupressaceae	Conifers	[3]
Maullinia_ectocarpii	Desmarestitales	Brown algae	[13]
Maullinia_ectocarpii	Ectocarpales	Brown algae	
Maullinia_ectocarpii	Ectocarpus	Brown algae	
Maullinia_ectocarpii	Laminaciales	Brown algae	
Maullinia_ectocarpii	Scythamnales	Brown algae	
Maullinia_sp	Durivillaea	Brown algae	[1]
Octomyxa breviglegnia	Brevilegnia	Oomycetes	
Octomyxa breviglegnia	Geolegnia	Oomycetes	
Octomyxa_achlyae	Achlya	Oomycetes	[14]
Phagomyxa_bellerocheae	Bellerochea	Diatoms	[15]
Phagomyxa_odontella	Odontella	Diatoms	
Sorodiscus_cokeri	Pythium	Oomycetes	[3]
Sorosphaerula_veronicae	Plantaginaceae	Eudicots	[3]
Woronina_glomeratea	Vaucheria	Crysophytes	[3]
Woronina_polycystis	Achlya	Oomycetes	[3, 16]
Woronina_polycystis	Aphanomyces	Oomycetes	
Woronina_polycystis	Apodachlya	Oomycetes	
Woronina_polycystis	Dictyuchus	Oomycetes	
Woronina_polycystis	Pythium	Oomycetes	
Woronina_polycystis	Saprolegnia	Oomycetes	
Woronina_polycystis	Thraustotheca	Oomycetes	
Woronina_pythii	Achlya	Oomycetes	[3, 17]
Woronina_pythii	Dictyuchus	Oomycetes	
Woronina_pythii	Pythium	Oomycetes	
Woronina_pythii	Saprolegnia	Oomycetes	
Woronina_pythii	Thraustotheca	Oomycetes	

Supplementary Tab. S3: List of all environmental sequences included in Fig S1, including the sampling site where they come from and a more detailed description of the subsample where they were derived from. The number of times sequences with a similarity higher than 98% was obtained in cloning libraries is given in the last column (# ID^{98} Seq). The names of the 18S-clades follow the numbering in Fig. 5. Clades 4 and 10 are not listed as these did not include any environmental sequences.

Clade	Sampling site	Subsample	Accession number	Sequence code	# ID^{98} Seq
Clade 1	Bernkastel, DE	*Veronica* sp. rhizosphere direct	KF111182	PD0104	1
		Poa sp. rhizosphere direct	KF111196	PD0200	1
		Taraxacum sp. rhizosphere direct	KF111198	PD0217	1
		Rumex sp. rhizosphere direct	KF111205	PD0322	2
		mixed rhizosphere clones	KF111186	PD0139	6
		Poa spp. rhizosphere clones	KF111225	PD0229	3
			KF111219	PD0002	7
			KF111164	PD0043	7
			KF111176	PD0082	8
		soil clones	KF111188	PD0152	9
			KF111187	PD0140	64
	Lohbach, AT	soil clones	KF111203	PD0253	3
			KF111204	PD0258	2
	Volga 2, RU	soil clones	KF111158	PD0022	1
			KF111171	PD0070	1
	Volga 3, RU	soil clones	KF111173	PD0077	2
			KF111178	PD0089	2
			KF111162	PD0041	4
	Volga 5, RU	soil clones	KF111153	PD0007	4
	Wehlen, DE	*Vitis* spp. rhizosphere	KF111233	PD0241	1
	South Africa 2_3, ZA	soil clones	KF111207	PD0398	1
			KF111208	PD0401	5
			KF111206	PD0396	9
	South Africa 3_5, ZA	soil clones	KF111211	PD0430	5
			KF111212	PD0434	11
	South Africa 3_6, ZA	soil clones	KF111213	PD0459	11
Clade 2	Rotmoos, AT	*Deschampsya* sp. rhizosphere direct	KF111167	PD0049	1
		Poa sp. rhizosphere direct	KF111193	PD0196	1
Clade 3	Bernkastel, DE	*Veronica* sp. rhizosphere direct	KF111218	PD0478	1
		Vitis sp. rhizosphere direct	KF111180	PD0099	1
Clade 5	Ukraine6, UA	*Vitis* sp. rhizosphere direct	KF111194	PD0197	1
			KF111177	PD0085	1
	Ukraine8, UA	*Vitis* sp. rhizosphere direct	KF111152	PD0005	1
	Uruguay6, UY	*Vitis* sp. rhizosphere direct	KF111156	PD0017	1
	Bernkastel, DE	mixed rhizoshphere clones	KF111184	PD0118	1
			KF111183	PD0117	2
		Chenopodium sp. rhizosphere direct	KF111181	PD0101	1
Clade 6	Rotmoos, AT	*Poa* sp. rhizosphere direct	KF111192	PD0195	1
			KF111159	PD0025	1
	Uruguay6, UY	*Vitis* sp. rhizosphere direct	KF111195	PD0199	1
Clade 7	Rotmoos, AT	*Juncus* sp. rhizosphere direct	KF111221	PD0013	1
Clade 8	South Africa 2_5, ZA	soil clones	KF111209	PD0408	6
Clade 9	Bernkastel, DE	*Trifolium* sp. rhizosphere clones	KF111155	PD0016	2
	Lohbach, AT	soil clone	KF111199	PD0244	2
			KF111201	PD0248	2
			KF111217	PD0477	2
			KF111200	PD0245	3
Clade 11	Volga 1, RU	soil clones	KF111174	PD0080	2
			KF111165	PD0044	6
Clade 12	South Africa 4, ZA	soil clones	KF111215	PD0463	6
			KF111216	PD0473	6
Clade 13	Bernkastel, DE	*Chenopodium* sp. rhizosphere direct	KF111197	PD0212	1
		soil clones	KF111190	PD0182	1
			KF111191	PD0191	2
			KF111189	PD0164	2
		Trifolium sp. rhizosphere clones	KF111172	PD0074	6
	Lohbach, AT	Soil clones	KF111202	PD0252	2
	Volga 1, RU	soil clones	KF111169	PD0056	1
	Volga 2, RU	soil clones	KF111160	PD0033	6
			KF111175	PD0081	2
	Volga 3, RU	soil clones	KF111157	PD0018	1
Clade 14	Volga 2, RU	soil clones	KF111170	PD0057	1
			KF111116	PD0045	5
Clade 15	Bernkastel, DE	*Trifolium* sp. rhizosphere clones	KF111154	PD0014	1
		mixed rhizosphere clones	KF111185	PD0120	1
	Rotmoos, AT	*Carex* sp. rhizosphere direct	KF111161	PD0037	1
	South Africa 4, ZA	soil clones	KF111214	PD0461	1
	South Africa 3_2, ZA	soil clones	KF111210	PD0414	15
Clade 16	Volga 4, RU	soil clones	KF111168	PD0054	1
			KF111163	PD0042	2

Supplementary Tab. S4: Overview of phytomyxid sequences from clone libraries or direct sequencing from different sampling sites. The total number of samples collected from each site is given along with the number of positive detections using the plasmodiophorid specific primer strategy (Samples/PCR positives). Note that each soil sample was at least processed in four parallels, but multiple positives from the same sample were pooled prior to cloning and sequencing. Names 18SrDNA types (Clades on Fig 5) which were found in the different clone libraries are listed following the taxonomy suggested in Fig 5.

Sample Site	Sample type	Habitat	Samples/PCR positives	Number of clone libraries	#18S-types @ 98%	Clades on Fig 5	Sample code	Genbank accession number
Vineyard, Bernkastel (Germany)	soil	Commercial vineyard	200/63	4	5	Clade 1	PD0152<br>PD0140	KF111188<br>KF111187
						Clade 13	PD0182<br>PD0191<br>PD0164	KF111190<br>KF111191<br>KF111189
	Roots, clone libraries	Commercial vineyard	141/69	4	11	Clade 1	PD0139<br>PD0043<br>PD0082<br>PD0094	KF111186<br>KF111164<br>KF111176<br>KF111179
						Clade 5	PD0118<br>PD0117	KF111184<br>KF111183
						Clade 9	PD0016	KF111155
						Clade 13	PD0074	KF111172
						Clade 15	PD0014<br>PD0120	KF111154<br>KF111185
	Roots direct sequencing	Commercial vineyard	See above	0	8	Clade 1	PD0104<br>PD0200<br>PD0217<br>PD0322	KF111182<br>KF111196<br>KF111198<br>KF111205
						Clade 3	PD0478<br>PD0099	KF111218<br>KF111180
						Clade 5	PD0101	KF111181
						Clade 13	PD0212	KF111197
Glacier forefield (Austria)	Roots, direct sequencing	Glacial sucession site	14/5	0	5	Clade 2	PD0049<br>PD0196	KF111167<br>KF111193
						Clade 6	PD0195<br>PD0025	KF111192<br>KF111159
						Clade 15	PD0037	KF111161
Flood plains (Russia)	Soil samples		5/5	5	16	Clade 1	PD0022<br>PD0070<br>PD0077<br>PD0089<br>PD0041<br>PD0007	KF111158<br>KF111171<br>KF111173<br>KF111178<br>KF111162<br>KF111153
						Clade 11	PD0080<br>PD0044	KF111174<br>KF111165
						Clade 13	PD0056<br>PD0033<br>PD0081<br>PD0018	KF111169<br>KF111160<br>KF111175<br>KF111157
						Clade 14	PD0057<br>PD0045	KF111170<br>KF111166
						Clade 16	PD0054<br>PD0042	KF111168<br>KF111163
Flood plains (Austria)	Soil samples	Small drainage ditch in former flood plain	1/1	2	7	Clade 1	PD0253<br>PD0258	KF111203<br>KF111204
						Clade 9	PD0244<br>PD0248<br>PD0477<br>PD0245	KF111199<br>KF111201<br>KF111217<br>KF111200
						Clade 13	PD0252	KF111202
Fynboss (South Africa)	Soil samples	South African fynboss flora soil samples	20/6	6	11	Clade 1	PD0398<br>PD0401<br>PD0369<br>PD0430<br>PD0434<br>PD0459	KF111207<br>KF111208<br>KF111206<br>KF111211<br>KF111212<br>KF111213
						Clade 8	PD0408	KF111209
						Clade 12	PD0463<br>PD0473	KF111215<br>KF111216
						Clade 15	PD0461<br>PD0414	KF111214<br>KF111210

Supplementary Tab. S5: Samples used in this study including the number of subsamples, and the Sample side code used to refer to these samples.

Sample Site	Sampling Date	Soil samples	Plant sample	Water samples	Sample site code
Vineyard (Germany)	23-May-2008	40	32	0	Bernkastel
Vineyard (Germany)	14-July-2008	40	29	0	Bernkastel
Vineyard (Germany)	6-October-2008	40	29	0	Bernkastel
Vineyard (Germany)	28-May-2009	40	22	0	Bernkastel
Vineyard (Germany)	1-August-2009	40	23	0	Bernkastel
Glacier forefield (Austria)	14-July-2010	4	15	1	Rotmoos
Agricultural site (Austria)	Aug/Sept 2010	2	0	2	Ampass
Flood plains (Russia)	Summer 2010	5	0	0	Volga 1 - Volga 5
Flood plains (Austria)	Summer 2010	1	0	0	Lohbach
Flood plains (Austria)	Summer 2011	1	0	0	Lohbach
Fynboss (South Africa)	December 2011	20	0	0	South Africa

References cited in Supplementary material

1. Goecke F, Wiese J, Nunez A, Labes A, Imhoff JF, Neuhauser S: **A Novel Phytomyxean Parasite Associated with Galls on the Bull-Kelp *Durvillaea antarctica* (Chamisso) Hariot.** *PLoS One* 2012, **7**.
2. Walker AK, Campbell J: **First Records of the seagrass parasite *Plasmodiophora diplantherae* from the northcentral Gulf of Mexico.** *Gulf Caribb Res* 2009, **21:**63-65.
3. Karling JS: *The Plasmodiophorales.* New York: Hafner Publishing Company; 1968.
4. Ludwig-Muller J, Bennett RN, Kiddle G, Ihmig S, Ruppel M, Hilgenberg W: **The host range of *Plasmodiophora brassicae* and its relationship to endogenous glucosinolate content.** *New Phytol* 1999, **141:**443-458.
5. Barr KJ, Asher MJC: **The Host Range of *Polymyxa betae* in Britain.** *Plant Pathol* 1992, **41:**64-68.
6. Hugo SA, Henry CM, Harju V: **The role of alternative hosts of *Polymyxa betae* in transmission of beet necrotic yellow vein virus (BNYVV) in England.** *Plant Pathol* 1996, **45:**662-666.
7. Desoignies N, Stocco C, Bragard C, Legreve A: **A new phenotype of *Polymyxa betae* in *Arabidopsis thaliana*.** *Eur J Plant Pathol* 2011, **131:**27-38.
8. Legreve A, Schmit J, Bragard C, Maraite H: **The role of climate and alternative hosts in the epidemiology of rhizomanina.** In *Symposium of the International Working Group on Plant Viruses with Fungal Vectors; Bologna, Italy*. Edited by Rush C. 2005
9. Legreve A, Vanpee B, Delfosse P, Maraite H: **Host range of tropical and sub-tropical isolates of *Polymyxa graminis*.** *Eur J Plant Pathol* 2000, **106:**379-389.
10. Huber L, Eisenbeis G, Ruhl E, Pagay V, Kirchmair M: **Distribution and host range of the grapevine plasmodiophorid Sorosphaera viticola.** *Vitis* 2007, **46:**23.
11. Andersen BAB, Nicolaisen M, Nielsen SL: **Alternative hosts for potato mop-top virus, genus Pomovirus and its vector *Spongospora subterranea* f.sp *subterranea*.** *Potato Research* 2002, **45:**37-43.
12. Qu X, Christ BJ: **The host range of *Spongospora subterranea* f. sp *subterranea* in the United States.** *American Journal of Potato Research* 2006, **83:**343-347.
13. Maier I, Parodi E, Westermeier R, Muller DG: ***Maullinia ectocarpii* gen. et sp nov (Plasmodiophorea), an intracellular parasite in *Ectocarpus siliculosus* (Ectocarpales, Phaeophyceae) and other filamentous brown algae.** *Protist* 2000, **151:**225-238.
14. Pendergrass WR: **Studies on a Plasmodiophoraceous Parasite, *Octomyxa brevilegniae*.** *Mycologia* 1950, **42:**279-289.
15. Schnepf E, Kühn SF, Bulman S: ***Phagomyxa bellerocheae* sp. nov. and *Phagomyxa odontellae* sp. nov., Plasmodiophoromycetes feeding on marine diatoms.** *Helgol Mar Res* 2000, **54:**237-241.
16. Goldie-Smith EK: **The Position of *Woronina polycystis* in the Plasmodiophoraceae.** *Am J Bot* 1954, **41:**441-448.
17. Dylewski DP, Miller CE: **Systematic and Host Range Studies of *Woronina pythii* (Plasmodiophoromycetes) and Host, *Pythium* Species, from Axenic Culture.** *Mycologia* 1983, **75:**412-422.
